# Simple and Versatile Turbidimetric Monitoring of Bacterial Growth in Liquid Cultures Using a Customized 3D Printed Culture Tube Holder and a Miniaturized Spectrophotometer: Application to Facultative and Strictly Anaerobic Bacteria

**DOI:** 10.3389/fmicb.2016.01381

**Published:** 2016-08-31

**Authors:** Margarida R. G. Maia, Sara Marques, Ana R. J. Cabrita, R. John Wallace, Gertrude Thompson, António J. M. Fonseca, Hugo M. Oliveira

**Affiliations:** ^1^REQUIMTE, LAQV, ICBAS, Instituto de Ciências Biomédicas de Abel Salazar, Universidade do PortoPorto, Portugal; ^2^REQUIMTE, LAQV, DGAOT, Faculdade de Ciências, Universidade do PortoPorto, Portugal; ^3^CIBIO, InBIO-Research Network in Biodiversity and Evolutionary Biology, Universidade do PortoVairão, Portugal; ^4^Departamento Clinicas Veterinárias – ICBAS, Instituto de Ciências Biomédicas de Abel Salazar, Universidade do PortoPorto, Portugal; ^5^Rowett Institute of Nutrition and Health, University of AberdeenAberdeen, UK

**Keywords:** bacterial growth, liquid culture, miniaturized spectrophotometer, optical density, 3D print

## Abstract

Here we introduce a novel strategy for turbidimetric monitoring of bacterial growth in liquid culture. The instrumentation comprises a light source, a customized 3D printed culture tube holder and a miniaturized spectrophotometer, connected through optical cables. Due to its small footprint and the possibility to operate with external light, bacterial growth was directly monitored from culture tubes in a simple and versatile fashion. This new portable measurement technique was used to monitor the growth of facultative (*Escherichia coli* ATCC/25922, and *Staphylococcus aureus* ATCC/29213) and strictly (*Butyrivibrio fibrisolvens* JW11, *Butyrivibrio proteoclasticus* P18, and *Propionibacterium acnes* DSMZ 1897) anaerobic bacteria. For *E. coli* and *S. aureus*, the growth rates calculated from normalized optical density values were compared with those ones obtained using a benchtop spectrophotometer without significant differences (*P* = 0.256). For the strictly anaerobic species, a high precision (relative standard deviation < 3.5%) was observed between replicates up to 48 h. Regarding its potential for customization, this manifold could accommodate further developments for customized turbidimetric monitoring, such as the use of light-emitting diodes as a light source or flow cells.

## Introduction

Monitoring bacterial cell growth provides valuable information about their nutritional and energetic physiology, as well as an understanding of the survival and proliferation conditions of different species under different conditions (Koch, 2010). To this end, different techniques such as direct counts (e.g., using optical microscopy and flow cytometry), colony counts, biomass measurement, or light scattering have been used (Koch, 2007). Light scattering, which is based on the deflection of light by individual cells, is the most convenient property to be measured, by quantifying either the light deflected or, more commonly, the turbidity of a culture in a spectrophotometer. Light passing through the culture reflects both the incident light deflected by the bacteria, together with deflected light that is diverted in a secondary fashion internally within the cultures back into the light path. The latter becomes of particular importance at higher cell densities (Lin et al., 2010; Myers et al., 2013). Turbidimetry has the advantage of being fast and non-destructive. Therefore, the determination of the turbidity, or optical density (OD), of liquid cultures may be considered the most widespread analytical tool to monitor the growth of pure bacterial cultures.

Over the last few decades, this measurement approach has benefited from the evolution of optical technology. Instruments that incorporate different light sources and detectors have been used for measuring the number of cells in different formats, from culture tubes to microplates (Matlock et al., 2011). For example, recent developments in this field have been based on the development of high-throughput assays (Mertens et al., 2012), and on the study of microbial colonies (Mertens et al., 2011), particularly applied to the food microbiology field (Lobete et al., 2015). On the other hand, the development of imaging techniques also allowed the implementation of the McFarland method by combining a simple digital camera and an open source software (Lahuerta Zamora and Perez-Gracia, 2012), or the monitoring of cultures at low densities using a laser source and a charge-coupled device (CCD) camera (Julou et al., 2012). The use of UV light sources to monitor bacterial growth is also another topic of growing interest (Park et al., 2012; Szermer-Olearnik et al., 2014).

Nevertheless, all the laboratory measurements up to date are essentially based on benchtop instruments with a relatively large footprint, which commonly require sample handling (e.g., dilution). This could be particularly cumbersome when pathogenic or anaerobic microorganisms are the targets of the measurement operation. Hence, the use of portable optical setups that could accommodate different formats of the sample holder (e.g., culture tubes, cuvettes, and flow cells), and that could be easily operated on bench and laminar flow chambers could contribute to faster, simpler, and safer monitoring of bacterial growth.

In this context, the advent of miniaturized spectrophoto meters (Kantzas et al., 2009; Pena-Pereira et al., 2011) and 3D printers (Campbell et al., 2011) created the conditions for the customization of the measurement of OD for different microbiological applications. Miniaturized CCD based spectrophotometers can provide high optical accuracy and reliability in the UV-VIS-NIR range, with a minimal footprint and power consumption. Moreover, it is possible to operate using external light, making these instruments portable and easy to use under different experimental conditions, including *in situ* analysis. On the other hand, 3D printing is creating new opportunities to manufacture new customized laboratory hardware with simple design workflows and reduced costs (Pearce, 2014).

Based on this background, we introduce here a new simple and portable setup for the rapid measurement of bacterial growth in culture tubes, by combining a commercial miniaturized spectrophotometer with a customized 3D printed tube holder. We applied this new manifold to the measurement of growth of pathogenic and rumen anaerobic bacteria, and compared the new approach to the classic measurement protocol based on benchtop spectrophotometers.

## Materials and Methods

### Reagents and Solutions

Aqueous solutions were prepared with ultra pure water (maximum conductivity of 0.055 μS cm^-1^) produced by a Sartorius arium pro water purification system (Goettingen, Germany). McFarland turbidity standards were prepared by mixing aliquots of a 1% aqueous barium chloride (Fluka, Buchs, Switzerland) with 1% sulphuric acid (Fisher Scientific, Waltham, MA, USA). A stock solution of 1000 mg L^-1^ of bromothymol blue (BTB) (Merck, Darmstad, Germany) was prepared from the dissolution of the solid dye in the appropriate volume of 0.1 mol L^-1^ boric acid (Chem-lab, Zedelgem, Belgium) at pH 9.5. The BTB working standards were prepared by stepwise dilution of the stock solution using the same buffer as solvent.

### Apparatus

**FIGURE 1:**
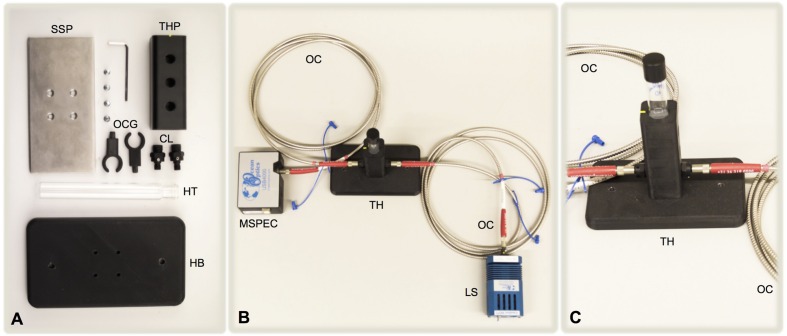
**Detailed parts of the 3D printed culture tube holder **(A)**, the miniaturized setup used for monitoring the bacterial growth **(B)**, and a detailed view of the tube holder **(C)**.** SSP, solid steel platform; THP, tube holder part; OCG, optical cable guides; CL, collimating lens; HT, Hungate tube; HB, holder’s base; MSPEC, miniaturized spectrophotometer; OC, optical cable; TH, tube holder; LS, light source. For sake of simplicity, the power connection to the light source and the power/data communication USB connection from the miniaturized spectrophotometer to the computer were not represented.

The customized tube holder (**Figure 1**) was designed using Solidworks 3D software (Dassault Systèmes Solidworks Corporation, MA, USA). The device was manufactured using a Fortus 250mc (Stratsys, Eden Prairie, MN, USA) 3D printer. ABSplus (Stratasys, Eden Prairie, MN, USA), a modified version acrylonitrile butadiene styrene (ABS) thermopolymer, was the material employed. A pair of 6 mm collimating lens (Sarspec, Vila Nova de Gaia, Portugal) with SMA (SubMiniature version A) connectors was placed 20 mm from the bottom of the holder (other distances in the same axis are also possible), and a solid steel platform was assembled to the holder’s base to improve its stability. Complete details about the tube holder parts and dimensions are available in the electronic Supplementary Material.

The detection system unit used in this work comprised a UV-VIS USB4000 miniaturized spectrophotometer controlled through the software Spectra Suite 2.0.162 (Ocean Optics, Dunedin, FL, USA), and a LS-1 visible light source (Ocean Optics). Light was conducted through a pair of 400 μm SMA terminated optical cables (QP-400-VIS-NIR-BX, Ocean Optics). For comparison purposes, a Jasco V-530 UV-Vis benchtop spectrophotometer (Tokyo, Japan) was also used to perform OD measurements in the selected growth experiments. In this case, 10 mm disposable plastic cuvettes were used. OD measurements were performed at 600 nm, and BTB standards were measured at 625 nm. The liquid broth was used as culture blank throughout the experiments.

### Bacterial Cultures

The pathogenic, facultative anaerobic bacteria *Escherichia coli* ATCC 25922 and *Staphylococcus aureus* ATCC 29213 were obtained from the General Hospital of Santo António – Centro Hospitalar do Porto (Porto, Portugal). Both bacteria were revived from -80°C, cultured once on 5% sheep blood agar (bioMérieux, Marcy l’Etoile, France) at 37°C for 24 h and pre-grown at 37°C overnight in pre-warmed Brain Heart Infusion Broth (BHI; Merck, Darmstadt, Germany) with shaking. These pre-grown cultures were diluted immediately before initiating the growth experiments (Harris et al., 2002; Lin et al., 2010).

Strictly anaerobic ruminal bacteria *Butyrivibrio fibrisolvens* JW11, *Butyrivibrio proteoclasticus* P18 and *Propionibacterium acnes* DSMZ 1897 were from the culture collection held at the Rowett Institute of Nutrition and Health (Aberdeen, UK). The type strain of *P. acnes* DSMZ 1897 was originally obtained from the Deutsche Sammlung von Mikroorganismen und Zellkulturen GmbH (Braunschweig, Germany) and *B. fibrisolvens* JW11 and *B. proteoclasticus* P18 were isolated from sheep (Wallace and Brammal, 1985; Wallace et al., 2006). All transfers and incubations were carried out under O_2_-free CO_2_ at 39°C in Hungate-type tubes (Hungate, 1969). Inoculum volumes were 5% (v/v) of a fresh culture into 10 mL of medium [liquid form of M2 medium (Hobson, 1969)].

### Growth Experiments

Both pathogenic bacteria suspensions were diluted in BHI in order to obtain a suitable initial OD for the experiments (0.01 to 0.05 at 600 nm). Triplicates of each bacteria were grown in BHI at 37°C in Erlenmeyer flasks shaken at 200 rpm and OD measurements were performed for 9 h with both spectrophotometers (Ocean Optics USB4000 and JASCO UV-Vis, corresponding to a miniaturized setup and benchtop instrument, respectively) until stationary phase was reached (Harris et al., 2002; Baev et al., 2006; Lin et al., 2010). With the anaerobic ruminal bacteria, inoculated culture tubes were incubated in duplicate in a water bath at 39°C. The OD was measured using the experimental manifold at 0, 1, 2, 4, 6, 8, 10, 12, 22, 24, 26, 28, 30, 32, 34, 36, 46, and 48 h.

### Statistics and Data Analysis

Bacterial growth curves were analyzed using the grofit R software package (Kahm et al., 2010). For *E. coli*, *S. aureus*, and McFarland standards, the relationship between the growth rate values from the growth curves (slope of calibration curve in the case of McFarland standards) obtained from the measurement using either the experimental device or the benchtop spectrophotometer were evaluated by using the PROC MIXED procedure of SAS (version 9.1, SAS Institute, Inc., Cary, NC, USA). Due to the different range of slope values found in each analytical instrument, the mixed model analysis was run with normalized values. The model included the fixed effects of slope and intercept, the random effect of culture, and the random residual error as described by St-Pierre (2001). An autoregressive covariance structure for the intercept and slopes was chosen according to the finite sample corrected Akaike information criterion and the Schwarz Bayesian information criterion (Wang and Goonewardene, 2004). Adjusted observations (slopes) were calculated by adding the residual from each individual observation to the predicted value of the study regression (St-Pierre, 2001). These adjusted observations were corrected for each culture (for the mixed model, McFarland standards were considered a culture).

## Results and Discussion

### Design of the Optical System for OD Measurement

Considering the objective of developing a simple and versatile system for the measurement of bacterial growth in liquid cultures based on their OD, we designed the tube holder (**Figure 1**) suitable for the use of commonly used anaerobic culture tubes (Hungate tubes) to perform the measurements. The holder incorporated a pair of collimating lenses (**Figure 1**) to focus the light beam toward the spectrophotometer. In order to increase the flexibility of the measurements, the lens pair can be moved among three different positions on the vertical axis. The different parts of the holder were 3D printed using ABSplus (additive manufacturing) and then assembled. The optical cables connected the light source to the holder, and the latter to the spectrophotometer.

This new monitoring instrument was used to establish calibration curves using McFarland and BTB standards, in order to individually evaluate the linearity of the scattering and absorption components of the OD measurement (Myers et al., 2013), respectively (**Table 1**). Hence, we found significant (*P* < 0.001) linear correlations (*r*^2^ > 0.99) between absorbance and McFarland standards up to McFarland 8, and a BTB concentration up to 60 mg L^-1^. These values corresponded to maximum absorbance values of ∼2.500 in both standard sets. For comparison purposes, the same standard solutions were measured in a benchtop UV-Vis double beam spectrophotometer. Similar linear ranges were observed for McFarland and BTB standards, but with maximum absorbance values of ∼1.500. Therefore, in our proposed miniaturized setup, the sensitivity of the measurement increased 1.8 times for McFarland standards and 1.4 times for BTB standards when compared with the benchtop spectrophotometer. The primary explanation for these differences is related to the optical path used associated with each instrument, which was ∼16 mm for the tubes (proposed setup) and 10 mm for the benchtop spectrophotometer, respectively. This enhanced sensitivity for either absorption or scattering components can impact on the separation of these two absorption components when necessary (Myers et al., 2013), since the miniaturized spectrophotometer is able to perform multi-wavelength measurements.

**Table 1:** Parameters of the calibration curves for McFarland and bromothymol blue (BTB) standards obtained using the miniaturized setup (miniaturized spectrophotometer) and benchtop UV-Vis spectrophotometer.

	Miniaturized setup		Benchtop spectrophotometer	
		
	McFarland	BTB	McFarland	BTB
Slope	0.326 ± 0.004	0.0353 ± 0.0001	0.181 ± 0.004	0.0252 ± 0.009
Intercept	-0.019 ± 0.042	0.001 ± 0.002	0.050 ± 0.020	0.045 ± 0.032
*r*^2^	0.998	0.999	0.995	0.997


### Measurement of Growth of Pathogenic Bacteria and Method Comparison

**FIGURE 2:**
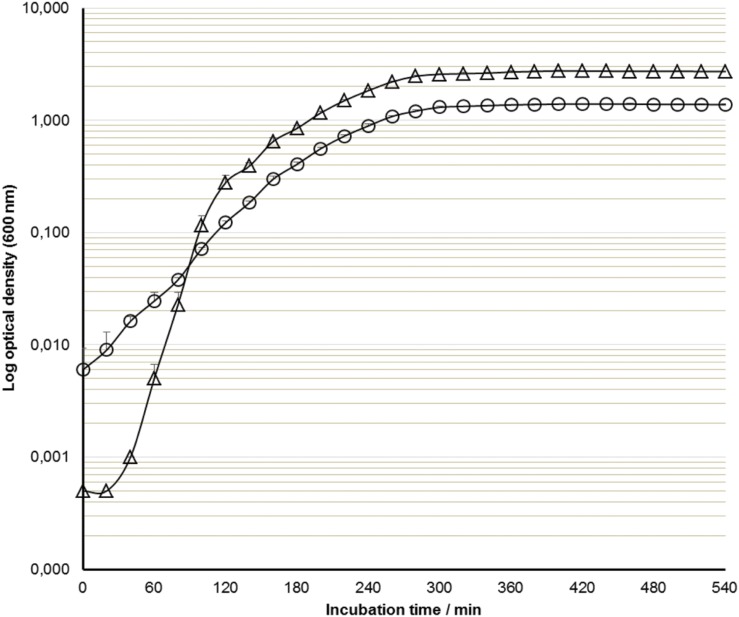
**Growth curves of *Escherichia coli* obtained by measuring the OD at 600 nm in the miniaturized setup (optical path of ∼16 mm) (triangles), and the benchtop UV-Vis spectrophotometer (optical path of 10 mm) (circles).** Results are means and standard deviation from three cultures.

Bacterial cells may have different light scattering angles according to their size that will directly impact on the OD values recorded (Koch and Ehrenfeld, 1968). Furthermore, the optical geometry of the spectrophotometer, especially optics collimation, may also impact on the measurements, since poor collimation leads to a higher percentage of scattered light that can reach the detector. This corresponds to lower absorbance values that result in linearity deviations of the measurements. In this context it was necessary to perform parallel measurements of OD in our miniaturized optical setup and also in a benchtop spectrophotometer in order to assess the potential use of the proposed methodology for routine measurement of bacterial growth. To this end, each *E. coli* and *S. aureus* cultures grown in Erlenmeyer flasks were then transferred to tubes and cuvettes, where OD was measured for nine consecutive hours. As shown in **Figure 2**, the use of these two optical configurations led to substantially different OD values for the same Erlenmeyer flask cultures of *E. coli*. Similar differences were observed for the growth curve of *S. aureus* (data not shown). These differences in the recorded OD values were in agreement with the results observed for the preliminary linearity studies with McFarland and BTB standards (**Table 1**), and can be primarily justified by the differences on the optical path of each instrument. Therefore, we hypothesized that the differences between the absolute OD values generated by the two optical setups were originated by the differences of the two instruments used, which could affect the measurements due to their different optical paths and/or optical geometry. In order to test our hypothesis, we modeled bacterial growth curves resorting to gcbootspline function (part of grofit R software package), and estimated the three characteristic parameters: specific growth rate (μ), length of lag phase (λ), and the maximum cell growth (A) for both bacterial suspensions measured using both spectrophotometric configurations (**Table 2**).

**Table 2:** Parameters of the growth curves of *Escherichia coli* and *Staphylococcus aureus* obtained using the miniaturized setup and a benchtop UV-Vis spectrophotometer.

Bacteria	Method	Replicate #	μ	λ	*A*
*E. coli*		1	0.944 ± 0.044	2.49 ± 1.04	2.591 ± 0.003
	Miniaturized	2	0.916 ± 0.052	2.66 ± 1.14	2.576 ± 0.003
		3	0.889 ± 0.073	2.55 ± 1.07	2.449 ± 0.002
		1	0.570 ± 0.024	2.72 ± 1.04	1.398 ± 0.001
	Benchtop	2	0.559 ± 0.032	2.62 ± 1.05	1.390 ± 0.003
		3	0.535 ± 0.049	2.91 ± 1.04	1.370 ± 0.002

*S. aureus*		1	0.719 ± 0.039	2.27 ± 1.37	2.590 ± 0.001
	Miniaturized	2	0.677 ± 0.046	2.08 ± 0.90	2.558 ± 0.014
		3	0.680 ± 0.023	1.88 ± 1.34	2.506 ± 0.003
		1	0.395 ± 0.015	2.30 ± 1.42	1.357 ± 0.003
	Benchtop	2	0.327 ± 0.010	2.42 ± 1.17	1.377 ± 0.007
		3	0.388 ± 0.019	2.39 ± 1.40	1.397 ± 0.005


*μ, growth rate; λ, length of the lag-phase; A, maximum cell growth.*

Although OD values differed when the two different spectrophotometers were used, the period of time corresponding to the different phases of the growth curves were identical (**Figure 2**). Thus, we focused on the differences between the μ values obtained using the different optical instrumentation because it reflects the balanced growth of a bacterial culture (Campbell, 1957; Schaechter, 2015). Moreover, it can be used for the comparison of different definitions of bacterial growth measurements (Koch, 2007). Therefore, we compared the normalized μ values of the growth curves of *E. coli* and *S. aureus* obtained in both measurements (miniaturized setup and benchtop), as well the slopes of the calibration curves obtained for the McFarland standards under the same measurement conditions. The mixed model analysis showed that the μ values were unaffected by the culture evaluated (*P* = 0.256). Furthermore, a significant relationship (*r*^2^ = 0.99, *P* = 0.004) was also observed between the μ values obtained using miniaturized and the benchtop spectrophotometer [miniaturized spectrophotometer μ = -0.034 (±0.0775) + 1.003 (±0.1183) × benchtop spectrophotometer μ]. These observations are in agreement with the comparative study performed by Matlock et al. (2011), where different UV-Vis spectrophotometers showed different OD values for bacterial cultures and McFarland standards due to the different optical geometries that are present in each instrument. The same authors also described that is possible to calculate a conversion factor between instruments that normalizes data obtained among different spectrophotometers. This was confirmed by the correlation found between the normalized μ values in both instruments used throughout this work. Nevertheless, this conversion factor is a property of each individual culture due to the different absorption and scattering characteristics of the individual cells (Lin et al., 2010; Myers et al., 2013). In the present case, we concluded that similar profiles could be found in both measurement conditions, showing that the miniaturized setup can be adopted as routine analytical tool for bacterial growth profiling.

### Measurement of Growth of Anaerobic Ruminal Bacteria

Another potential application of the measurement strategy proposed in this work concerns the growth of strictly anaerobic bacteria. The growth monitoring of these microorganisms is difficult due to the mandatory manipulation of the culture under anaerobic conditions and the concomitant interference of oxygen. Hence, we evaluated the feasibility of our methodology to monitor the growth of three anaerobic ruminal species - *B. fibrisolvens*, *B. proteoclasticus*, and *P. acnes* – in Hungate culture tubes. After measuring the OD values for 48 h, we modeled the growth curves using the gcFit function (part of the grofit R package; Kahm et al., 2010) that estimated the abovementioned parameters of the growth curves: μ, λ, A (**Table 3**). In this case, we found a high repeatability between the two different culture tubes prepared from the same inoculum. For all parameters of the growth curves, the relative standard deviation (RSD) values were below 3.5% between the two replicates. This enhanced precision is a key characteristic for the routine profiling of bacterial growth based on the OD of the liquid culture. The setup is also useful in this particular situation, considering that the culture tubes of the anaerobic species studied here must be kept in an O_2_-free atmosphere (Stewart et al., 1997).

**Table 3:** Parameters of the growth curves *Propionibacterium acnes*, *Butyrivibrio fibrisolvens*, *Butyrivibrio proteoclasticus*, obtained using the miniaturized setup.

Bacteria	Model	μ	λ	*A*
*P. acnes*	Richards	0.0860 ± 0.0027	8.42 ± 0.51	1.452 ± 0.010
		0.0862 ± 0.0037	8.27 ± 0.70	1.456 ± 0.012
*B. fibrisolvens*	Logistic	0.387 ± 0.043	7.07 ± 0.23	1.468 ± 0.016
		0.376 ± 0.048	6.99 ± 0.27	1.460 ± 0.018
*B. proteoclasticus*	Logistic	0.257 ± 0.034	7.67 ± 0.41	1.566 ± 0.021
		0.245 ± 0.032	7.59 ± 0.39	1.558 ± 0.020


*μ, growth rate; λ, length of the lag-phase; A, maximum cell growth.*

### Comparison with Other OD Measurement Approaches

In UV-Vis spectrophotometers, OD is a light scattering measurement translated by an absorbance value, with the consequence that the use of different instruments commonly leads to different results due to the different optical configurations present (Matlock et al., 2011; Myers et al., 2013). Typical UV-Vis benchtop spectrophotometers for OD measurement comprise microplate and dual-beam formats that usually ensure a suitable sensitivity and linear range. However, they also require the transfer of the liquid culture to a microplate or a cuvette or a dilution protocol (Matlock et al., 2011; Myers et al., 2013), which presents several constraints when pathogenic or anaerobic bacteria have to be measured. In contrast, the setup proposed here was able to bypass sample handling when the bacteria are directly cultured into the tubes (this was the case with the rumen bacteria), and can also be used in appropriate places for sample manipulation (e.g., laminar flow hoods) due to its portability. Additionally, it is also possible to measure OD at different wavelengths and/or the complete absorption spectrum of the sample. This also impacts in the growth profiling of species or media that have a strong absorption component (Matlock et al., 2011; Myers et al., 2013) or when other light scattering sources may be present (Hernández and Marín, 2002; Bernardez and De Andrade Lima, 2015).

Following this validation of the novel instrumentation, future development could take advantage of further developments in 3D printing solutions (Gross et al., 2014), LED light sources (Macka et al., 2014), and flow cell designs (Tymecki et al., 2008; Myers et al., 2013), which would lead to new online monitoring tools for the accurate and precise measurement of bacterial growth.

## Conclusion

This research paper introduces a simple and versatile strategy for the monitoring of bacterial growth, by combining a customized 3D culture tube holder with a miniaturized spectrophotometer. This made possible the monitoring of bacterial growth in a portable fashion, and with a minimal or no sample handling. This solution is particularly suitable for direct reading of culture tubes of pathogenic and anaerobic microorganisms, where handling has to be minimized or avoided. The assembling of a pair of collimating lenses to the holder minimized the dispersion of the light caused by the circular surface of the glass tube, generating growth profiles similar to those ones obtained using a dual-beam benchtop spectrophotometer. Regarding its modular character, this solution can also accommodate new customizations able to fulfill each individual user’s requirements.

## Author Contributions

HO conceived and designed the study. MM, SM, and HO conducted the experiments. HO, AC, RW, and AF analyzed the data and interpreted the results in collaboration with MM and SM. HO drafted the manuscript with contributions by all authors. GT edited the manuscript. All authors gave final approval for publication.

## Conflict of Interest Statement

The authors declare that the research was conducted in the absence of any commercial or financial relationships that could be construed as a potential conflict of interest.
