# Supplementary material for: Simple and Versatile Turbidimetric Monitoring of Bacterial Growth in Liquid Cultures Using a Customized 3D Printed Culture Tube Holder and a Miniaturized Spectrophotometer: Application to Facultative and Strictly Anaerobic Bacteria
Source: Front Microbiol. 2016 Aug 31;7:1381. doi: 10.3389/fmicb.2016.01381 (PMC5006086; doi:10.3389/fmicb.2016.01381)

Supplementary Material

Simple and versatile turbidimetric monitoring of bacterial growth in liquid cultures using a customized 3D printed culture tube holder and a miniaturized spectrophotometer: application to facultative and strictly anaerobic bacteria

Margarida R. G. Maia, Sara Marques, Ana R. J. Cabrita, R. John Wallace, Gertrude Thompson, António J. M. Fonseca, Hugo M. Oliveira*

*** Correspondence:** Hugo M. Oliveira: hmoliveira@icbas.up.pt

Exploded view and dimension details of the culture tube holder. 1- collimating lens, 2- anti-slip rubber, 3- cuture tube, 4- stainless steel platform, 5- base, 6- mounting screws, 7- tube holder part with three mounting places for the collimating lenses. All dimensions in millimeters.

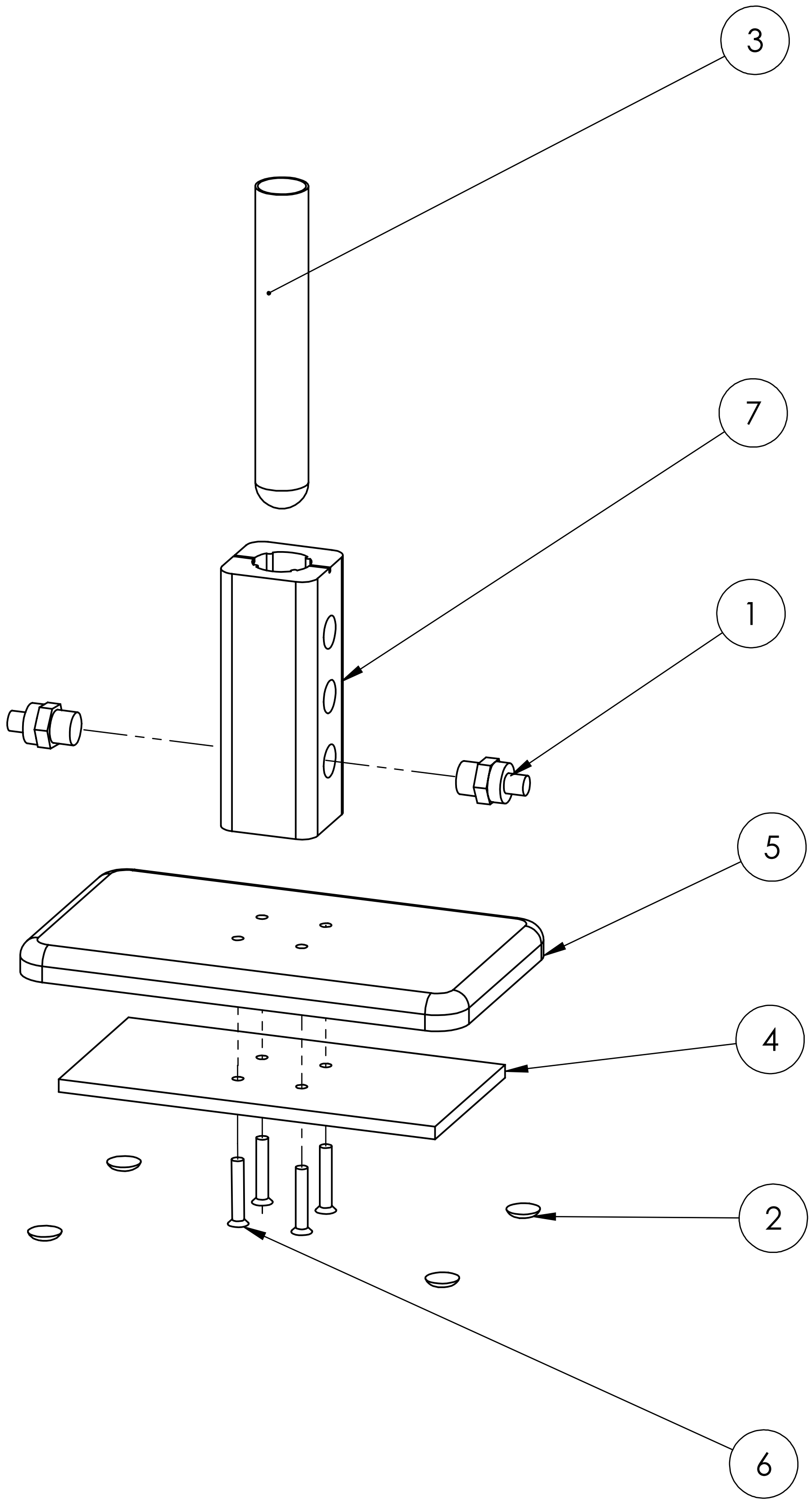
3
7
1
5
4
2
6

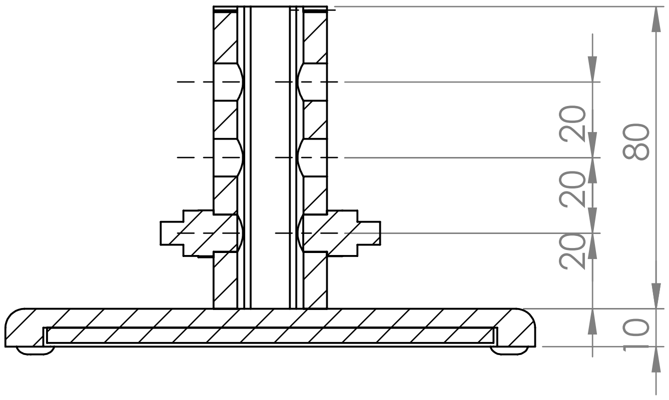

SECTION A-A

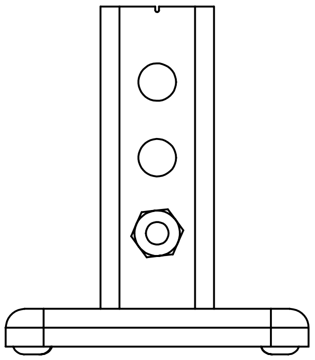

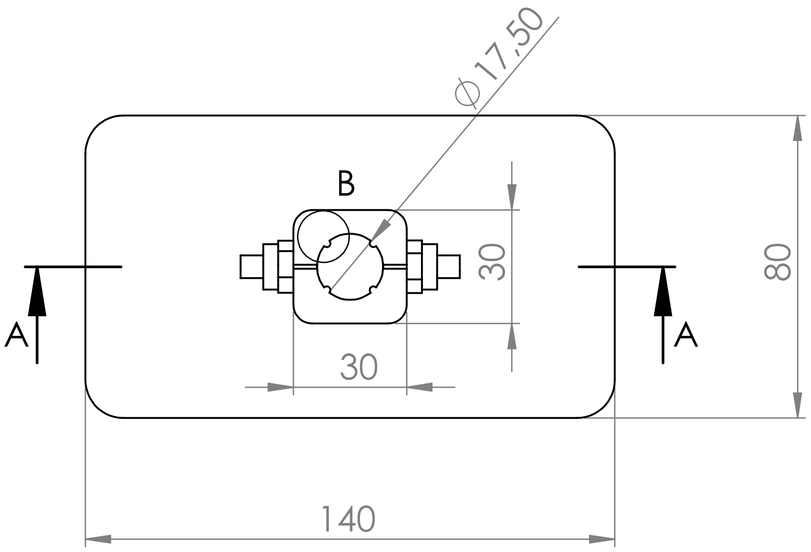

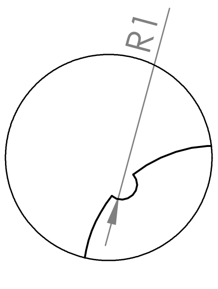

DETAIL B
SCALE 2 : 1

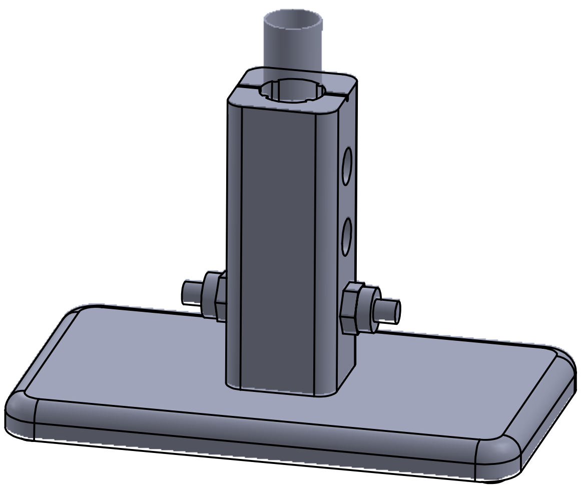

Supplement: Supplementary file 1 [file Image_1.PDF]
